# Comparative RNA-Seq Analysis Reveals Macrophage Polarization and T Cell Exhaustion Signatures in Visceral Leishmaniasis

**DOI:** 10.3390/ijms27125425

**Published:** 2026-06-16

**Authors:** Rohit Raj, Priya Kumari, Abhik Sen, Manas Ranjan Dikhit

**Affiliations:** ICMR-Rajendra Memorial Research Institute of Medical Sciences, Agamkuan, Patna 800007, India; rohitrajcuh@gmail.com (R.R.); priyakumari2166@gmail.com (P.K.); abhik.sen@icmr.gov.in (A.S.)

**Keywords:** *Leishmania* pathogenesis, VL, *L. donovani*, macrophage polarization

## Abstract

The Syrian golden hamster (*Mesocricetus auratus*) is a universally accepted model for visceral leishmaniasis (VL) due to its ability to mimic human disease pathology. *Mus musculus* (BALB/c) is preferred for evaluating pharmaceutical and immunological responses. This study focuses on the precise role of gene signatures in *L. donovani*-infected *M. auratus* and *M. musculus*, using transcriptomic analysis. Principal component analysis (PCA) revealed distinct clustering among the four groups (uninfected vs. infected spleen samples from *M. auratus* and *M. musculus*). After differential expression analysis, 2054 genes in *M. auratus* and 1108 in *M. musculus* were found to be differentially expressed, with 153 genes common to both species. Except for 31 genes, most of the commonly dysregulated genes show a similar expression pattern. Although Th1-mediated immune signaling was observed in both cases, the overexpression of *LAG3* in both infected groups underscores the important role of T cell exhaustion. Immunological responses against parasite infection in *M. auratus* appear to be more aggressive, while *M. musculus* seems more intense. Interestingly, only the *M. musculus*-infected group shows overexpression of *IL-10*. Without a definitive role for *IL-10*, the overexpression of *Tgm2, Clec7a*, and *Adora2b* in both species may drive disease outcome. These findings elucidate the immunological mechanisms driving the pathogenesis of VL in rodent models.

## 1. Introduction

VL poses a substantial public health challenge in tropical and subtropical regions, particularly in South Asia, East Africa, and South America. The primary pathogen is *L. donovani*, transmitted by sandfly bites [1]. VL mainly affects the poor, where people have little or no access to health facilities [2]. The disease is associated with a myriad of clinical symptoms, from fever to more advanced stages like anemia and hepatosplenomegaly. Unfortunately, VL diagnosis and treatment are difficult due to the vague initial symptoms, which, if untreated, worsen over time [3,4]. A core component of VL pathology is immune evasion, which allows the host *Leishmania* parasites to remain undetected during infection [5,6,7]. The parasite employs intricate mechanisms to alter host immune cell activity, particularly modulating cytokine and chemokine expression to create a favorable environment. Evidence shows that during VL infection, *Leishmania* downregulates the production of pro-inflammatory cytokines, such as TNF-α and IL-12, which are crucial for macrophage activation and parasite clearance [3,8,9]. Such a blockage enables parasites to survive and propagate throughout the organism.

Simultaneously, *Leishmania* modulated the host immune system to release the anti-inflammatory cytokines, such as IL-10, which dampen immune responses and help parasite survival [9]. The parasites also modify antigen presentation by altering surface molecules, such as lipophosphoglycan (LPG), leading to T-cell immunological unresponsiveness and subsequent immune evasion [4]. Furthermore, *Leishmania* strategically resides within intracellular compartments such as the parasitophorous vacuole to avoid host immune effectors and ensure prolonged survival [10]. The complex interactions between hosts and pathogens, particularly in VL, require an in-depth examination of gene expression patterns. Although the spleen is central to the body’s immune response, in the case of an infection with *Leishmania* parasites, very little information persists [11]. Inflammatory mediators are associated with an augmented inflammatory response and assist in the persistence and transmission of the parasites to other regions of the host [12]. Notably, the early stages of infection and the host’s immune cell activation in response to the invading *Leishmania* are noteworthy. To control the infection, pro-inflammatory cytokines such as TNF-α, IFN-γ, and IL-12 are known to be over-expressed [13]. At the same time, increased chemokine expression, such as CXCL10, attracts immune cells to the infection site, helping to contain the parasite [14].

Splenic CD4+ T cells exhibit a mixed effector-regulatory function and contain inhibitory receptors that hinder the action of macrophage effectors in progressive VL [11]. The development of the disease is aided by this dysregulated immune response, and transcriptome profiling in experimental VL reveals a wide range of inflammatory milieu in the spleen, which shapes macrophages into a disease-promoting phenotype. The overexpression of inflammatory mediators intensifies the inflammatory response, thereby promoting the survival and spread of parasites within the host [13]. The dual RNA-Seq technique sheds light on the shared transcriptional host responses observed in the spleens and livers of mouse models of VL caused by *Leishmania infantum* and *L. donovani*. Here, it highlights the up regulation of genes in the complement cascade, as well as in interferon gamma (ifn-γ) signaling pathways. Irrespective of the parasite species, the host immune responses against parasite invasion are usually the same [14]. These results highlight the complex interactions between immune cells and *Leishmania* parasites in the spleen during VL pathogenesis.

*Leishmania* parasites have several strategies to overcome host defense mechanism and modulate host cell gene expression to create a favorable microenvironment for the parasite [3]. Understanding these intricate gene expression dynamics during early pathogenesis is critical for elucidating the disease’s progression and for developing targeted interventions to mitigate its impact on public health.

## 2. Results

### 2.1. Dataset Description

**Figure 1:**
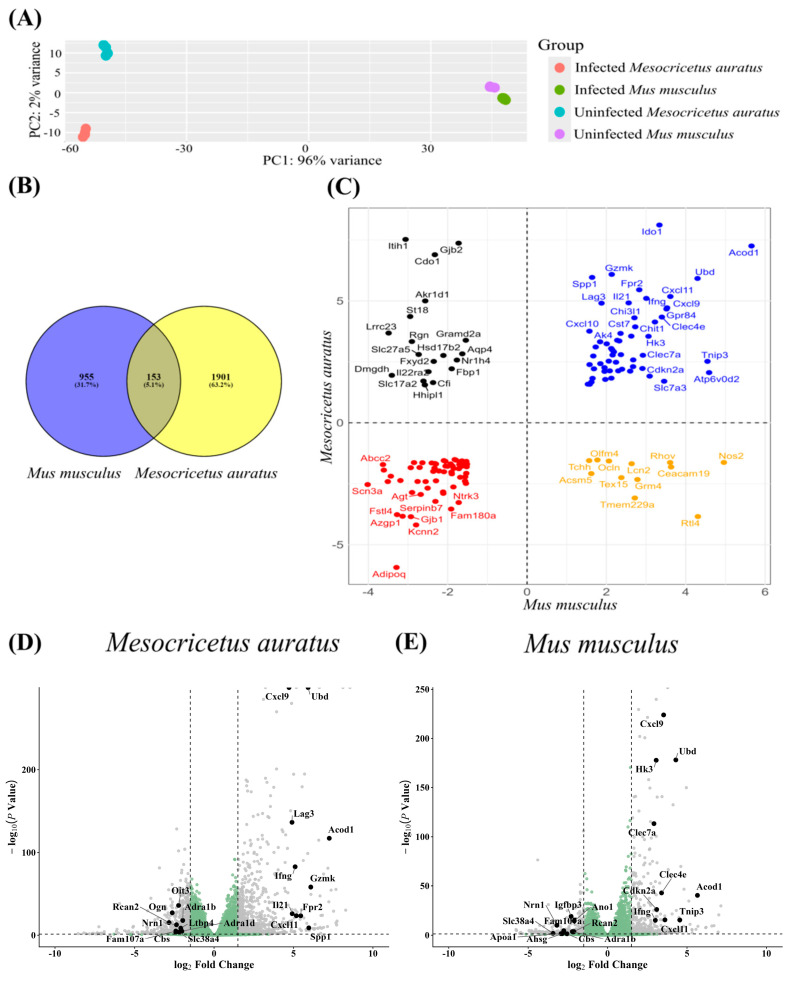
(**A**) Principal component analysis (PCA) of spleen RNA-seq samples from *M. auratus* and *Mus musculus*. Each dot represents one biological replicate. The number of spleen samples analyzed was *n* = 4 for *M. auratus* and *n* = 5 for *Mus musculus*, for both infected and uninfected conditions. Percent variance explained by each principal component is indicated on the axes. (**B**) Venn diagram indicating overlap among differentially expressed genes in *M. musculus* and *M. auratus*. (**C**) Quadrant plot for the entire 153 common gene across the *M. musculus* and *M. auratus*. Quadrant 1: gene in blue color that has log2fc ≥ 1.5 in both the mouse models; Quadrant 2: genes in black color are those which have log2fc ≥ 1.5 in *M. auratus* and log2fc ≤ −1.5 in *M. musculus*; Quadrant 3: genes in red color are those which have log2fc ≤ −1.5 in both the mouse models; and Quadrant 4: genes in yellow color are those which have log2fc ≥ 1.5 in *M. musculus* and log2fc ≤ −1.5 in *M. auratus*. (**D**) Volcano plots showing common differentially expressed genes in *M. musculus* and (**E**) *M. auratus*. Grey dots represent significantly differentially expressed genes. The common top 10 up-regulated and top 10 down-regulated genes, selected based on absolute log2 fold change, are highlighted in black and labeled with their corresponding gene symbols.

A comparative transcriptomic analysis was conducted on splenic tissues from *Leishmana donovani*-infected *M. musculus* and *M. auratus* models, compared with healthy controls. The main goal was to identify patterns in gene expression related to the infection in both species. For the *M. auratus* dataset, it was observed that the average read count was 53.31 million, which reduced to 49.81 million after trimming. In the case of *M. musculus*, the initial read count was 15.57 million, which reduced to 14.46 million after trimming. Normalization of the samples has to be performed to allow for the comparison since there is significant variation in the depth of sequencing among the species. This was addressed using DESeq2 size factor normalization. This technique calculates size factors for each sample based on the median ratio of a gene’s count to its geometric mean across all samples [15,16]. These factors were applied to the read counts to adjust for differences in sequencing depth. This allows for the assesment of gene expression levels within samples without accounting for changes in sequencing depth. With DESeq2 normalization, we reduced the impact of confounding variables related to sequencing depth, ensuring no bias in the subsequent differential expression analysis [16,17]. PCA performed on the 17-sample dataset indicates that independent clustering was present within the four experimental groups, i.e., infected and uninfected spleen samples from *M. auratus* and *M. musculus*, which demonstrates that the variation in the dataset was explained largely by a limited number of principal components. This enabled differentiation of the groups based on gene expression (Figure 1A).

### 2.2. Differentially Expressed Genes

**Figure 2:**
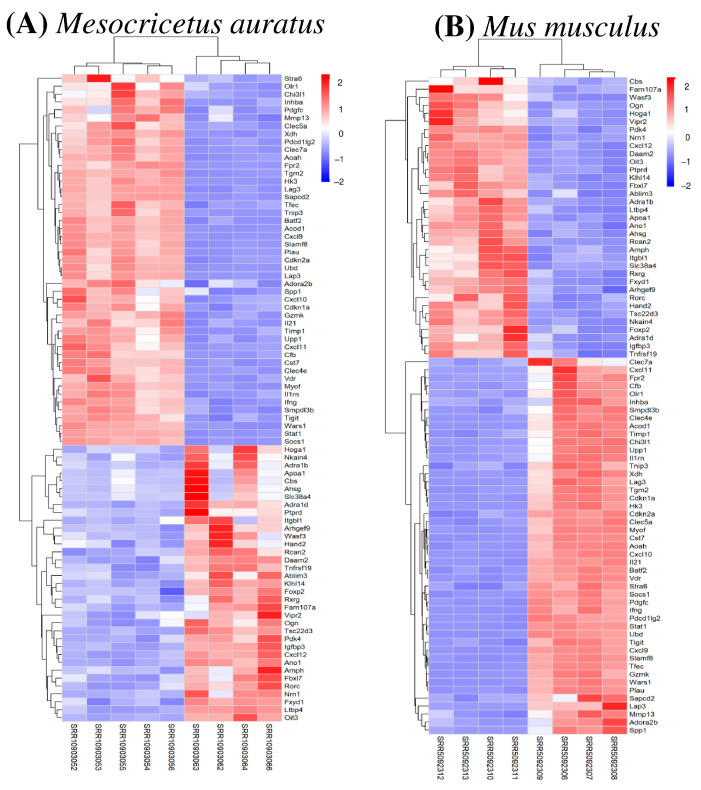
A heat map depicting the common differentially expressed genes (DEGs) in the spleen during *L. donovani* infection is shown for *M. auratus* (**A**) and *M. musculus* (**B**). Red indicates high expression values, while blue indicates low expression values upon scaling. Some samples exhibit slightly irregular expression compared to their co-group samples.

Differential expression analysis for the two species was conducted separately. For each species, the infected group was compared with its respective control group, i.e., *M. auratus* infected vs. *M. auratus* control, and *M. musculus* infected vs. *M. musculus* control. The commonly differentially expressed genes between the two species were selected for further analysis. After analyzing annotated differentially expressed genes (DEGs) in *M. auratus* with a Log2FC ± 1.5 filter and a *p*-value cutoff of 0.05, a total of 2054 genes were identified as differentially expressed. Of these genes, 1066 were found to be down-regulated, and 988 were found to be overexpressed. *M. musculus*, on the other hand, showed differential expression in 1108 genes, with 674 down-regulated and 434 up-regulated. It is noteworthy that *M. musculus* and *M. auratus* shared 153 (5.1%) differentially expressed genes (Figure 1B). Out of this set, 122 genes were found to have the same expression changes, which means they were either up-regulated or down-regulated in both species. Interestingly, 31 genes showed a complex differential expression, i.e., up-regulated in one species and down-regulated in the other, or vice versa (Figure 1C). This suggests an intricate immune signaling response to *L. donovani* infection across different animal models. The volcano plots reveal the distribution differentially expressed genes of both *M. auratus* (Figure 1D) and *M. musculus* (Figure 1E), with significantly altered genes shown in grey dots, while the top 10 up-regulated and down-regulated genes are shown with black dots, along with their gene symbols at a Log2FC ± 1.5 and *p* value cutoff 0.05. All genes that had a normalized count of fewer than two in at least three out of five samples were discarded from further analysis, leaving a subset of 82 genes for further investigation. These 82 genes showed consistent upregulation and downregulation patterns between *M. musculus* and *M. auratus*. The normalized expression levels of each gene across individual samples are displayed in the heatmap of the 82 frequently dysregulated genes (Figure 2).

### 2.3. Gene Ontology and Pathway Analysis

**Figure 3:**
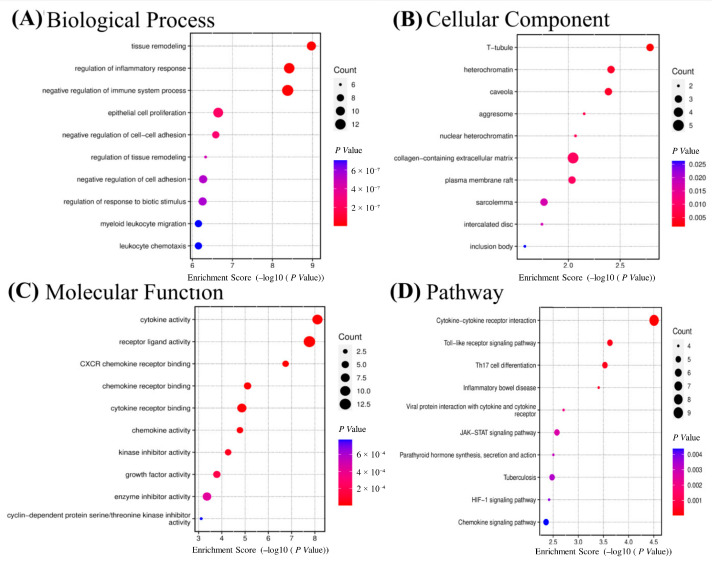
Gene ontology analysis of common DEGs in *M. musculus* and *M. auratus* derived spleen tissues. Bubble plots showing genes enriched in biological process (**A**), cellular components (**B**) and molecular function (**C**). Bubble plot for pathway analysis (**D**). The color represents *p* value and the size of the dot represents the number of genes.

Using the SR plot to perform Gene Ontology analysis identified distinct functional groups that were significantly enriched among the translated DEGs. These were biological constructs related to the control of tissue remodeling, control of inflammation, negative regulation of immune system processes, negative regulation of epithelial cell proliferation and cell–cell adhesion, negative regulation of tissue remodeling and cell adhesion, regulation of response to biotic stimulus, myeloid leukocyte migration, and leukocyte chemotaxis. Alongside this, enriched molecular functions included activity of a cytokine, activity of a receptor ligand, binding of CXCR chemokine receptor, binding of a chemokine receptor, binding of a cytokine receptor, chemokine activity, and kinase activity as a growth factor, and as an enzyme of inhibition and as an inhibitor of cyclin-dependent protein serine/threonine kinase. There was also significant enrichment in cellular components such as T-tubule, heterochromatin, caveola, aggresome, nuclear heterochromatin, collagen-containing extracellular matrix, plasma membrane raft, sarcolemma, intercalated disk, and inclusion body. The dot plots allowed for visual integration and interpretation of the enriched GO terms, which emphasize biological processes, cellular compartment, and molecular function (Figure 3A–C).

Moreover, pathway analysis identified the interconnected molecular pathways and cellular processes that are dysregulated under certain conditions. The enriched pathways among DEGs included cytokine–cytokine receptor interaction, toll-like receptor signaling pathway, Th17 cell differentiation, inflammatory bowel disease, viral protein interaction with cytokine and cytokine receptor, JAK-STAT signaling pathway, parathyroid hormone synthesis, secretion, and action, tuberculosis, HIF-1 signaling pathway, and chemokine signaling pathway (Figure 3D).

### 2.4. PPI and Identified Hub Gene

An 82-gene set of commonly dysregulated genes was used for Protein–Protein Interaction (PPI) network analysis. The STRING database in Cytoscape was employed to construct the interaction network. From this network, the largest connected subnetwork, comprising 47 nodes (genes) and edges (interactions), was extracted for the identification of hub genes (Figure S1). Notably, 20 genes emerged as prominent hub nodes within this network and were present in more than six of the 12 parameter topologies analyzed. Among these hubs, five genes, namely *Batf2*, *Cxcl9*, *Il1rn*, *Spp1*, and *Timp1*, exhibited an exceptionally high degree of connectivity (with a degree value of 11). Furthermore, two genes (*Cxcr3* and *Ifnγ*) showed a degree of 10, whereas five genes (*Cxcl10*, *Cxcl12*, *Lag3*, *Pdcd1lg2*, and *Stat1*) showed a degree of nine. Similarly, another subset of five genes also exhibited a degree of eight, and finally, three genes (*Mmp13*, *Tigit*, *Igfbp3*) demonstrated a degree of seven within the network.

**Figure 4:**
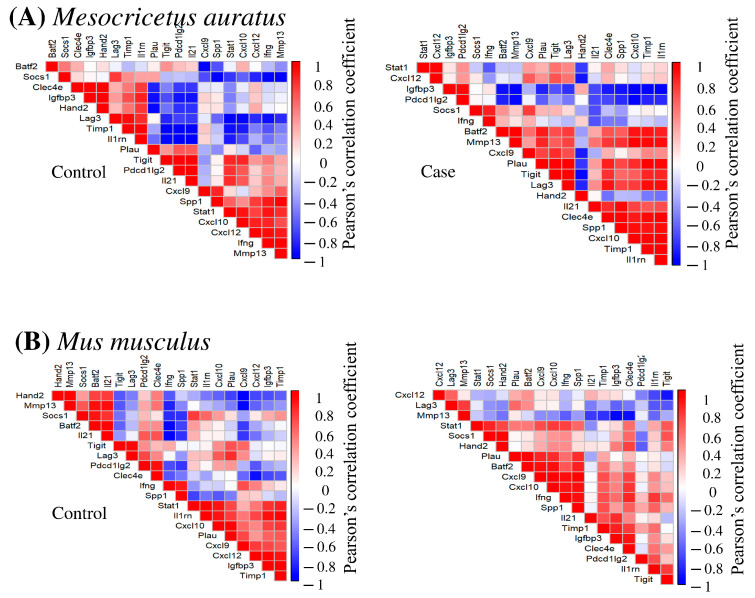
Correlation matrices within identified hub gene with VL in *M. auratus* (control and case; (**A**)) and *M. musculus* (control and case; (**B**)). Increasing values are represented by colors: red for positive correlation and blue for negative correlation. Significance: *p* < 0.05. Significant positive and negative correlations were observed among hub genes in diseased and healthy groups in *M. auratus*, such as *Stat1-Cxcl12* (r = 0.95, *p* = 0.047) and *Igfbp3-Timp1* (r = −0.97, *p* = 0.03). In *M. musculus*, strong correlations were noted, including *Cxcl9-Batf2* (r = 0.91, *p* = 0.03) and *Cxcl9-Spp1* (r = 0.96, *p* = 0.009), highlighting conserved and species-specific immune regulatory pathways in leishmaniasis.

Pearson correlation analyses of gene expression data from both *M. auratus* and *M. musculus* reveal significant correlations among hub genes in the infected and control groups, providing additional information about molecular interactions during leishmaniasis. In *M. auratus*, significant positive and negative correlations were seen, including *Stat1* and *Cxcl12* (r = 0.95, *p* = 0.047), *Igfbp3* and *Timp1* (r = −0.97, *p* = 0.03). This suggests that Stat1, which plays a role in immune modulation, and Cxcl12, a chemokine, are likely to be involved in modulating immune pathways in leishmaniasis [18]. Furthermore, in *M. musculus*, *Cxcl9* and *Batf2* (r = 0.91, *p* = 0.03), and *Cxcl9* and *Spp1* (r = 0.96, *p* = 0.009) were also significantly correlated. This indicates that chemokines and processes related to chemotaxis and immune cell activation, both critical to the disease process, are regulated by these genes [19] (Figure 4).

## 3. Discussion

The patho-physiology of *Leishmania* has been shown to differ across various animal models. In this article, we present a comparative analysis of gene expression profiles in *M. musculus* and *M. auratus* following infection with *L. donovani*. The results from using the GO term (GO:0002683) on the synonymous dataset indicate that negative regulation of the immune system may play a critical role in pathogenesis by creating a splenic immunosuppressive environment. Overall, even though some immune system responses were noted through the activities of cytokines, cytokine receptors, chemokines, and chemokine receptors. The activity of the kinases and the enzyme inhibitor response showed that they could inhibit the parasite’s response to host cell signaling or intentionally displace the macrophage antimicrobial response. These findings suggest that the parasite uses host signaling as a survival technique. Enrichment in tuberculosis-associated pathways suggests shared mechanisms of immune evasion with other intracellular pathogens. Furthermore, the observed increase in myeloid leukocyte migration and chemotaxis indicates that *Leishmana* potentially modulates immune cell movement to promote its dissemination and evade host immune responses [20]. The analysis showed that an increase in structures such as the T-tubule and sarcolemma at the cellular level possibly indicates disruptions in muscle and cardiac cell structures, which are thought to be systemic effects of VL [21].

Distinctive differences in expression fold change patterns of *CCL5* and *CXCL11* were observed among the two different species (*CCL5*; *M. musculus*: 0.39, *M. auratus*: 3.93, and *CXCL11*; *M. musculus*: 3.61, *M. auratus*: 5.18). This data indicates an augmented chemo-tactic response and enhanced immune cell recruitment in *M. auratus*. In both species, elevated levels of *IFN-γ* (*M. musculus* = 3.01; *M. auratus* = 5.10) suggest that a vigorous Th1 immune response persists against the parasitic infection. In contrast, *IL-10* expression levels exhibited divergent trends in *M. musculus* (2.05-fold increase) and *M. auratus* (0.05-fold increase). *IL-10*, a well-established anti-inflammatory cytokine, plays a critical role in regulating the immune response [22]. Elevated *IL-10* levels in *M. musculus* indicate a potentially enhanced capacity for immune regulation, whereas reduced *IL-10* expression in *M. auratus* suggests a diminished anti-inflammatory response, potentially resulting in heightened pro-inflammatory activity and immune activation. In these models, variations in *IL-10* expression may affect VL susceptibility, disease progression, and resolution. The relatively strong anti-inflammatory response of *IL-10* in *M. musculus* might help the host’s immune system by reducing inflammation and the impact of parasites.

On the other hand, *M. musculus* showed a fold change of −0.39 and *M. auratus* 3.46 for *CXCR6* expression, which showed an inverse pattern. These differences could be due to differences in how immune cells move and settle in tissues across species. Moreover, the infected spleens in both Th1-type cytokine models showed increased levels of chemokines, including *IL-21, CXCL9*, and *CXCL10*. *IL-21* overexpression may increase T-bet and *STAT4* expression in T cells, thereby increasing *IFN-γ* production [23]. On the other hand, both innate and adaptive immune responses to *Leishmania* infection depend on *CXCL9* (monokine induced by *IFN-γ, MIG*), *CXCL10* (IFN-γ inducible protein 10, IP-10), and *CXCL11* [24]. These chemokines target cells expressing the *CXCR3* receptor, including activated and memory CD4+ and CD8+ T cells, NK cells, macrophages, and certain dendritic cell subsets [25]. Th1 cell-mediated immunity is vital for combating leishmaniasis by modulating Th1 immune cells. However, despite increased chemokine expression during initial infection, they failed to control parasite replication. Studies have shown that genetically resistant mice lacking the *CXCR3* gene could generate an effective Th1 response in draining lymph nodes but could not control parasite growth at the lesion site due to defective T cell migration [26]. Previous studies have also indicated that, despite a significant increase in *IFN-γ* expression, *IFN-γ* has minimal impact on *Leishmana* survival [27,28]. Notably, *LAG-3* overexpression in infected conditions indicates a proactive role in controlling T cell activation and growth [29]. Possibly, this T cell exhaustion sets up a favorable environment for the parasite survival [30].

M2 macrophages play a critical role in mediating non-healing infection, even within a Th1 immune environment. Even though the Th1 immune response is positively associated with macrophage activation and effective control of infections, the presence of high numbers of tissue remodeling and anti-inflammatory M2 macrophages creates an environment in which parasites can persist and evade immune detection, leading to chronic infections. A better understanding of macrophage polarization and how *Leishmania* species exploit it may help develop countermeasures against leishmaniasis [31]. Overexpression of any of the following genes can significantly impair the pathogenesis of *Leishmana*: *Tgm2*, *Cdkn1a*, *Timp1*, *Clec5a*, *Clec4e*, *Plau*, *Batf2*, *Ubd*, *Tigit*, *Lag3*, *Vdr*, *Chi3l1*, *Adora2b*, and *Clec7a*. The gene in question is key to immune evasion, modification of host cell responses, and establishment of infection. The functions of such genes concerning the parasite’s survivorship and pathogenesis are a valuable domain for exploring novel therapeutic options. Transglutaminase 2 (TGM2) is a key enzyme that modifies proteins by catalyzing crosslinking reactions with low-molecular-weight primary amines.

Research by Martinez et al. (2013) highlighted the genetic programs expressed in resting and *IL-4* alternatively activated mouse and human macrophages, revealing similarities and differences [32]. Yamaguchi et al. (2016) revealed that *PLA2G5* controls the transglutaminase activity of human *IL-4*-activated M2 macrophages by generating *PGE2* [33]. Our analysis suggests that *TGM2* overexpression could activate macrophages and thereby support parasite survival. A similar function is conferred by Clec4e (Mincle) overexpression, which favors the parasite’s survival. Research on Mincle (macrophage-inducible C-type lectin, also known as *Clec4e* or *Clecsf9*) deficient mice (−/−), which lack the Mincle receptor, has shown enhanced immunity against Leishmania infections [34]. More than threefold overexpression of *Clec4e* in parasite-infected groups indicates the favorable role of *Clec4e* in parasite survival. Specifically, the parasites manipulate Mincle signaling, shifting it toward an inhibitory FcRγ/SHP1 axis in DCs, and these inhibitory signals help the parasites evade detection and clearance by the host’s immune system [35]. Similarly, overexpression of *Clec7a* (Dectin-1) indicated that immature dendritic cells (DCs), which express high levels of surface Dectin-1, preferentially infect Dectin-1+ myeloid cells with *Leishmania.* A previous study also indicated that, unlike macrophages, curdlan-exposed DCs alter Dectin-1 expression, potentially due to receptor internalization [36]. *Leishmania major* does not bind to Dectin-1, thereby preventing Dectin-1 signaling, DC maturation, and effective T-cell activation [36]. The correlation between CLECs and A2B receptors often involves their collective impact on immune responses. CLECs can influence the expression and activity of various receptors, including A2B, by modulating the immune environment. A direct correlation was observed between the up-regulation of A2B expression on monocytes and an increased parasite load. It was also demonstrated that activation of the A2B receptor is crucial for adenosine’s stimulatory effect on *IL-10* production and its suppression of nitric oxide release. The adenosine-induced *IL-10* production in *L. donovani*-infected macrophages was found to require ERK1/2 activation while being independent of p38 MAPK activation [37]. Therefore, the overexpression of the A2B receptor appears to alter macrophage function, thereby supporting the parasite’s survival.

This in-depth analysis explored the multifaceted interactions between host immune mechanisms and parasitic infections, utilizing two different rodent models. Differences in gene expression and immune responses were observed, suggesting very little species-specific variation. In both models, induced levels of *IFN-γ* expression demonstrated a vigorous Th1-dominant response. The altered *IL-10* expression in only one model suggests that parasite survival within the host is beyond the scope of *IL-10* expression. Notably, consistent gene interactions between *M. auratus* and *M. musculus*, such as *Cxcl9* and *Spp1*, demonstrate the persistence of pathways regulating the immune response during leishmaniasis. Both *Cxcl9* and *Spp1* facilitate the recruitment of immune cells that initiate local inflammation. Evidence thus far suggests they may serve as biomarkers of disease progression. The overexpression of *Stat1* and *Cxcl12* in *M. auratus*, as well as *Batf2* and Ifn-γ in *M. musculus* suggests that *Stat1* and *Batf2* likely have distinct immune regulatory functions. Stat1 is a notable transcription factor in the *IFN-γ* signaling pathway, enabling macrophage activation to control pathogens, while *Batf2* is significant in T-helper Cell differentiation pathways, suggesting model-specific immune pathways associated with the disease state [38]. Both *Batf2* and *Ifn-γ* (associated with *M. musculus*) were associated with Th1-dominant T-helper lymphocytes, representing model-specific immune pathways associated with infection. The gene expression analysis methodologies across species also reveal significant differences that require exceptionally rigorous quality control processes to ensure reliable comparative interpretation. Collectively, the findings of the differential expression of associated chemokines and cytokines that were of interest, specifically *CXCL11* and *IL-10*, suggest that the species assessed possess different cellular recruitment and inflammatory modulation abilities. This may affect disease presentation and resolution, as well as host susceptibility.

Our in-depth transcriptome analysis of murine and hamster models explores a clear immune landscape and demonstrates the importance of M2 macrophage polarization and T cell exhaustion in *Leishmana* pathogenesis. In addition to immune modulation and evasion, genes such as *Tgm2, Clec4e*, and *Adora2b*, which are involved in immune evasion and modulation, were identified, offering potential mechanistic processes that support parasite survival. A relatively small sample size may limit statistical power and detection of subtle expression changes; however, strict quality control, conservative statistics, and consistent replicate patterns support the robustness of the transcriptional signatures. Further validation of these signature markers across rodent models will help elucidate the underlying disease dynamics more clearly.

## 4. Methodology

### 4.1. Data Sources

The sequencing datasets of *M. auratus* with Accession No: GSE91187 and *M. musculus* with Accession No: GSE143799 were obtained from the NCBI GEO database (https://www.ncbi.nlm.nih.gov/gds/) accessed on 5 March 2024. The dataset GSE91187 comprises four *L. donovani*–infected and four uninfected spleen samples from *M. auratus*, whereas GSE143799 accessed on consists of five infected and five uninfected spleen samples from *Mus musculus*. The sequencing was carried out on the Illumina HiSeq 1000 and 2500 platforms, USA. The experiments provide useful tools to study gene expression patterns associated with *L. donovani* infection within the spleen and will better inform future investigations into the molecular mechanisms underlying the patho-physiology of VL.

### 4.2. Read Processing and Differential Gene Expression Analysis

The raw FASTQ files were preprocessed with FastQC v0.11.9 [39] to evaluate the general quality of the sequence, distribution of read lengths and adapter contamination. One *M. musculus* (SRR10903065) sample showed significantly lower sequencing depth as compared to the other samples. Due to the low read count, this sample was categorized as an outlier and removed from further analyses. Adapter sequences and low-quality reads were subsequently removed using Trimmomatic v0.36 [40]. Trimming was performed using the following parameters: minimum Phred quality score of 30, sliding window trimming with a window size of 4 and a required average quality score of 15, and a minimum read length of 120 bp after trimming. After that, HISAT2 v2.2.1 was used to align the reads to the reference genomes of *M. musculus* (GRCm39) and *M. auratus* (BCM_Maur_2.0) [41]. Alignments were performed using default parameters with splice-aware mapping enabled. Gene annotation files (GTF format) corresponding to each reference genome were provided to guide spliced alignment. Feature Counts were subsequently utilized to count the transcripts of the genes [42] and differential gene expression was conducted with DESeq2 v1.42.0 [15], with a *p*-value threshold of <0.05 and fold change threshold of ≥1.5. Principal component analysis (PCA) post-variance stabilizing transformation (VST) was performed on normalized counts of unique genes using R’s 4.3.1 DESeq2 tools. To account for inter-study batch effects, datasets were analyzed independently, and integration was performed at the level of shared differentially expressed genes rather than merged expression matrices. The DEGs were then subjected to gene ontology (GO) term enrichment analysis using SRplot [43] which explore the overexpressed reads in biological processes, molecular functions, and cellular components.

### 4.3. PPI Construction and Hub Gene Identification

Using the STRING (Search Tool for the Retrieval of Interacting Genes) database for Cytoscape v3.10.2, the 82 DEGs (differentially expressed genes) between two species of mouse models were constructed as protein–protein interaction (PPI) networks [44]. The following were kept as default: an interaction score of 0.4 (medium confidence), FDR (False Discovery Rate) of 5%, and the complete STRING network. Interactions were made based on a combination of evidence from the interaction databases, validated interactions, curation, the co-expression, and the text mining and computation prediction.

Hub genes were found through Cytoscape’s CytoHubba plug-in, which facilitates the network-centered prioritization of genes through a variety of topological computation methods. Out of 12 features, betweenness, bottleneck, closeness, clustering coefficient, degree, DMNC (Densest Maximum Neighborhood Component), eccentricity, EPC (Edge Percolated Component), MCC (Maximal Clique Centrality), MNC (Maximal Neighborhood Component), radiality, and stress were selected. Because each algorithm encapsulates different features of network topology, the combination of multiple metrics alleviates bias introduced by reliance on a single centrality measure and enables the robust identification of biologically meaningful hub genes, as described before [45,46]. For every topological parameter, genes were independently ranked, and 20 were selected. The ranking overlap was quantified by counting the number of ranked lists of genes that contained a gene and assigning a value to the gene for the number of ranked lists it was found on. Those ranked lists were considered high confidence hub targets. The identified hub targets genes were further correlated in the remaining resources, comparing the control and infected groups for *M. auratus* and *M. musculus* by Pearson correlation. Matrices were computed with the cor function, and significance was tested with the cor.test function at *p* = 0.05.

## Data Availability

The sequencing datasets of *M. auratus* with Accession No: PRJNA356860 and *M. musculus* with Accession No: PRJNA601732 were obtained from the NCBI GEO database (https://www.ncbi.nlm.nih.gov/gds/, accessed on 24 November 2025).

## Supplementary Materials

The following supporting information can be downloaded at https://www.mdpi.com/article/10.3390/ijms27125425/s1.
