# Supplementary figures and images for: Comparative RNA-Seq Analysis Reveals Macrophage Polarization and T Cell Exhaustion Signatures in Visceral Leishmaniasis
Source: Int J Mol Sci. 2026 Jun 16;27(12):5425. doi: 10.3390/ijms27125425 (PMC13299894; doi:10.3390/ijms27125425)

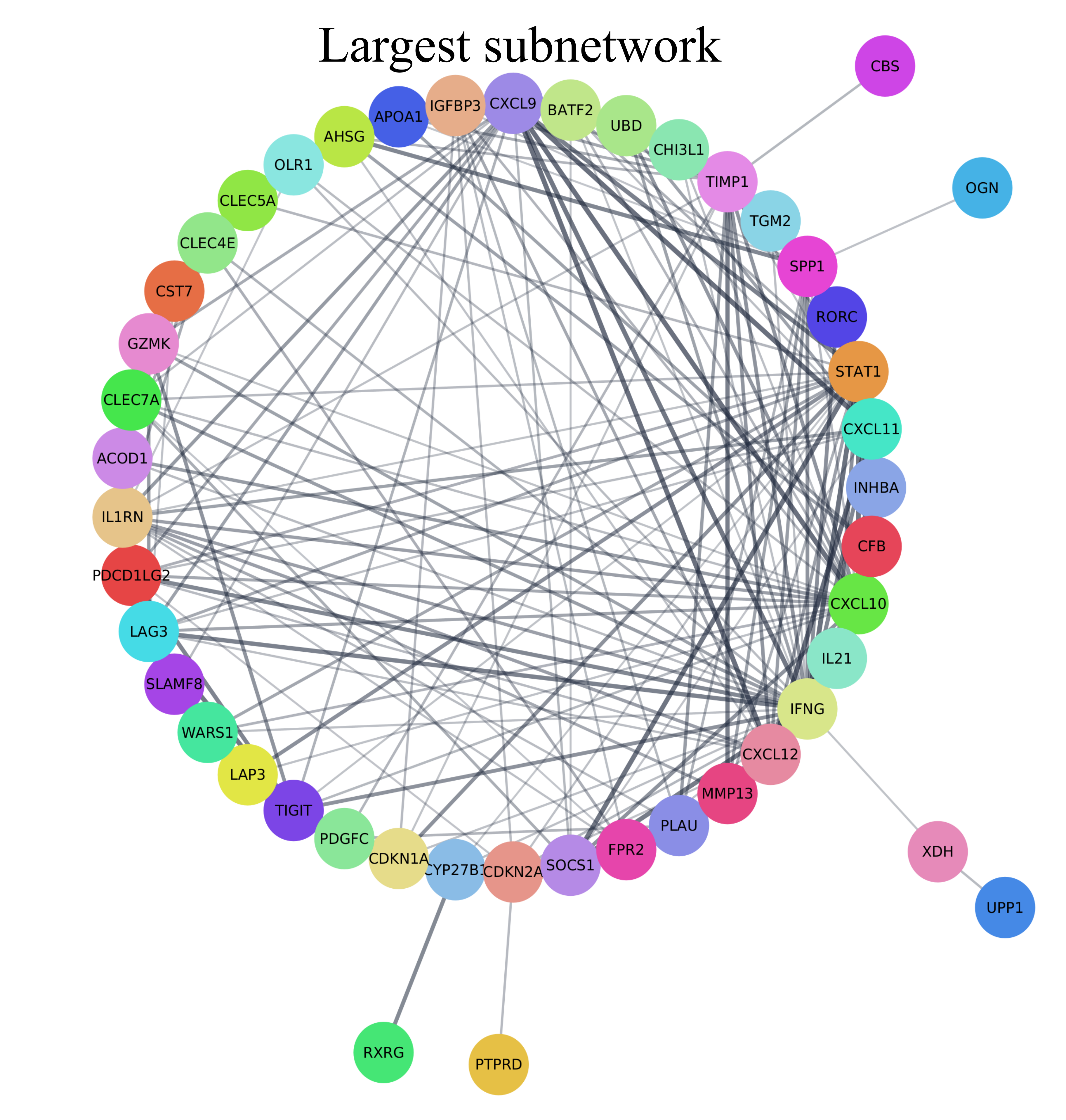

Supplement: Supplementary file 1 [file ijms-27-05425-s001.zip › ijms-4186273-supplementary.png]
